# Using auriculotherapy for osteoarthritic knee among elders: a double-blinded randomised feasibility study

**DOI:** 10.1186/s12906-016-1242-6

**Published:** 2016-07-29

**Authors:** Lorna K. P. Suen, Chao Hsing Yeh, Simon K. W. Yeung

**Affiliations:** 1 School of Nursing, The Hong Kong Polytechnic University, HungHom, Hong Kong; 2 Johns Hopkins School of Nursing, Room 421, 525, N. Wolfe Street, Baltimore, MD21205 USA

**Keywords:** Auriculotherapy, Osteoarthritic knee, Elderly, Magnetotherapy, Laser

## Abstract

**Background:**

Osteoarthritic knee (OA knee) is a common condition in the elderly. Exploration of non-invasive complementary therapies for OA knee is warranted given the limitations of pharmacologic therapies. Auriculotherapy (AT) is a therapeutic method in which specific points on the auricle are stimulated to treat various disorders of the body, and the therapeutic value and synergistic effect of laser auriculotherapy (LAT) when combined with magneto-auriculotherapy (MAT) merits further investigation.

**Methods:**

This study adopted a double-blinded four-arm randomized placebo design. The aims of study are (1) to assess the feasibility of AT among elders with OA knee in a future large-scale study, including the use of blinding in subjects and evaluators, acceptance of treatment protocol, and estimating the effect size and attrition rate; and (2) to evaluate the preliminary effect of AT in elders with OA knee. Subjects were randomly divided into four groups with different modes of AT with/without placebo objects. A total of 43 subjects completed the 6-week intervention and post-assessment. Assessments included a numerical rating scale of pain (NRS), the timed-up-and-go test (TUGT), and standard goniometer measurements during knee flexion and extension, Kruskal–Wallis test was used to evaluate differences among groups, and Wilcoxon sign-ranked test for examining within-group comparison.

**Results:**

Preliminary results indicated the absence of differences in the NRS, TUGT, and active/passive knee flexion and extension at baseline, as well as post-therapy, between the four groups. Even though the differences of these parameters between groups were not significant, the relative differences of NRS and TUGT in subjects who received combined MAT plus LAT were higher than those treated with MAT or LAT alone, or the placebo group. Four of the six parameters demonstrated significant within group differences in subjects who received MAT and/or LAT, whereas no significant differences were found in the placebo group.

**Conclusion:**

This study demonstrates that the AT protocol adopted in this study for elders with OA knee is feasible and could be applied in future larger-scale study. Larger sample size should be considered in a future trial to determine the causal relationship between treatment and effect.

**Trial Registration:**

ClinicalTrials.gov: NCT02352636. Registered on 23 January 2015.

## Background

Osteoarthritic knee (OA knee) is a common condition in the elderly [1] and accounts for more than 80 % of osteoarthritis cases [2]. Some sufferers may develop severe pain and impaired physical functions [1] as the disease progresses. Non-steroidal anti-inflammatory drugs mainly focus on musculoskeletal pain relief but often result in side effects, such as gastrointestinal haemorrhage [3]. For example, the use of acupuncture, which employs needles as a source of stimulation, may not be acceptable to people who suffer from needle phobia and may induce a risk of acupuncture-transmitted infections, such as acupuncture mycobacteriosis, through the transmission of microbes from acupuncturists to patients or patient-to-patient transmission [4]. Thus, other non-invasive complementary methods with less side effects for OA knee should be explored.

Auriculotherapy (AT), one of the approaches in traditional Chinese medicine (TCM), is a therapeutic method in which specific points on the auricle are stimulated to treat various disorders in the body [5]. This technique is a specialised form of acupuncture. in which the ear is viewed as a microsystem of the body [6]. In AT, different materials, such as acupuncture needles, plant seeds, magnetic pellets, or low-energy laser, are applied on acupoints of the ears for therapeutic purposes. The effectiveness of using magnetic pellets for AT (MAT) may be associated with the interaction of magnetic fields with body tissues, thereby resulting in functional changes in the body [7]. In contrast, laser AT (LAT) provides a simple and non-invasive alternative to needle acupuncture [8]. This technique has been widely used to treat medical conditions, such as alleviating musculoskeletal pain [9, 10], and insomnia [11, 12]. Laser is non-invasive and painless and presents no risk of infection and cross infection [8]. Thus, the therapeutic value and synergistic effect of laser when combined with MAT merits further investigation. The therapeutic effect is inferred for optimisation by the application of MAT after LAT because the former approach could offer continuous stimulation of acupoints after laser treatment as long as the magnet pellets on the ears are in situ.

This study was performed to assess the feasibility of applying AT to elders with OA knee in a future large-scale study. The use of blinding in subjects and evaluators, acceptance of treatment protocol, estimation of the extent of effect and attrition rate was evaluated. The preliminary effects of MAT, LAT or a combined approach on elders with OA knee were determined.

## Methods

### Settings and participants

This study is a double-blinded randomised placebo trial with four groups. Subjects who are 60 years of age or older were recruited from two elderly centres in Hong Kong by convenience sampling. The existing OA condition was assessed through physical examination with reference to the clinical criteria of the American College of Rheumatology [13]. Participants were recruited if they experienced knee pain and any three of the following: (1) 60 years of age or older; (2) ≤30 min of morning stiffness; (3) crepitus on active joint motion; (4) bony tenderness; (5) bony enlargement; or (6) no palpable joint warmth. Exclusion criteria were as follows: (1) other connective tissue diseases affecting the knee; (2) knee joint steroid injections within the preceding three months; (3) with a hearing aid or pacemaker in situ (this criterion was to avoid possible interaction between the devices and magnetic pellets); (4) received AT within the preceding three months; (5) suffering from aural injuries or infections and (6) inability to understand instructions or provide consent.

#### Interventions

**Fig. 1:**
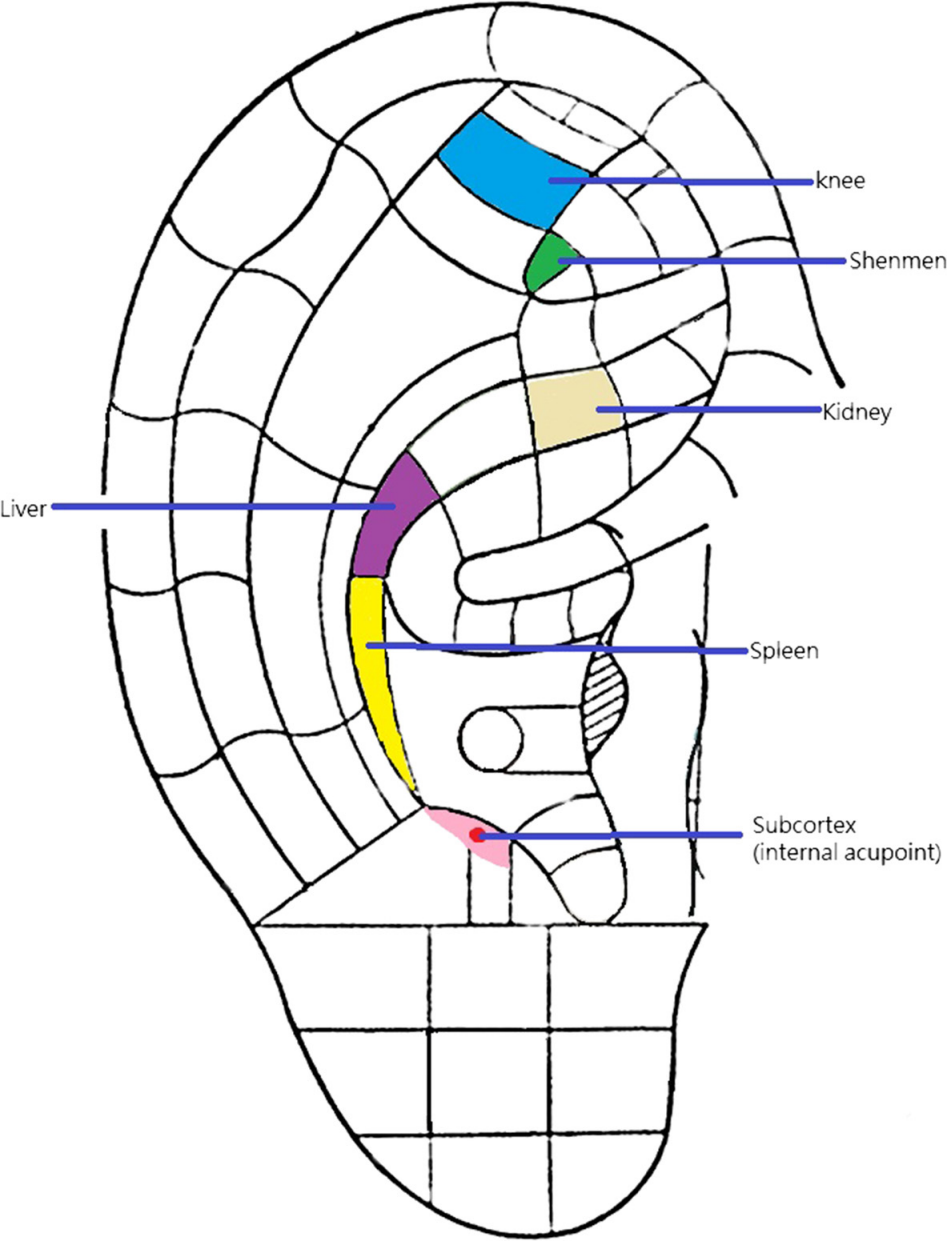
Selected ear acupoints for elders with osteoarthritic knee

Eligible subjects were randomly and blindly allocated to one of the four groups by a computer-generated randomised table. The random allocation sequence was managed independently, and random coding was concealed from participants and the evaluator until all data analyses have been completed. Six auricular acupoints which were inferred to have an effect on OA knee were selected. These acupoints included ‘shenmen’ (TF_4_), ‘knee’ (AH_4_), ‘spleen’ (CO_13_), ‘liver’ (CO_12_), ‘kidney’ (CO_10_) and ‘subcortex’ (AT_4_) [14]. The Chinese Standard Ear Acupoint Chart, which is recognised by the World Health Organization, and the latest nomenclature and location of auricular points announced by the China Standardization Organizing Committee in 2008 (GB/T 13734-2008) [15] was used to locate the acupoints (Fig. 1). The selection of the six auricular acupoints under study was based on traditional Chinese medical theory (for ‘shenmen’, ‘liver’, ‘spleen’ and ‘kidney’) and modern medicine (for ‘knee’ and ‘subcortex’). For example, ‘shenmen’ (TF_4_) is the main point for relieving pain. ‘Knee’ (AH_4_) is the local point for OA knee. ‘Liver’ (CO_12_) and ‘spleen’ (CO_13_) are for nourishing and strengthening the tendons and muscles, respectively, and ‘kidney’ (CO_10_) governs bones. ‘Subcortex’ (AT_4_) regulates the function of the central nervous system and induces sedative and pain relieving effects [14].

##### Group 1 (Placebo LAT and MAT)

The laser device was switched to ‘power off’ mode (that is, deactivated laser) for acupoint ‘stimulation’ prior to the application of MAT to achieve a blinding and placebo effect of the subject. Subjects were asked to wear a pair of laser protective goggles to ‘blind’ them during therapy administration. Thereafter, the magnetic pellets were applied to each selected acupoint. Each magnetic pellet, which contained an average of ~200 gauss/pellet magnetic flux densities and a diameter of 1.76 mm, was used.

##### Group 2 (LAT and placebo MAT)

Subjects received LAT via a laser device (pointer pulse). This device provides a wavelength of 650 nm, average output power of 2.5 mW, energy density of 1 min with 0.54 J/cm^2^ and a pulse of 10 Hz, which is a common, acceptable dosage for clinical use [9, 16]. A 1 min treatment using the continuous mode of the device was directly applied to the reactive region of each of the six selected acupoints on the ear. Laser protective goggles were provided to the subjects and researchers for eye protection. Similarly, a plaster without magnetic pellets that mimicked MAT treatment was applied on each acupoint after LAT.

##### Group 3 (LAT plus MAT)

Subjects received a combined approach using MAT plus LAT. LAT was administered prior to the application of MAT on selected acupoints, which would be implemented similarly to the procedures in groups 1 and 2.

##### Group 4 (Placebo)

Subjects served as a placebo control and received LAT at ‘power off’ mode (that is, deactivated laser) for acupoint ‘stimulation’ before the application of plaster without magnetic pellets that mimicked the MAT treatment.

The therapy was administered by a member of the staff who had received intensive coaching by the research team. The following procedures were standardised across the four groups to enhance the blinding effect of the subjects. The auricle of the participant was cleaned using 75 % isopropyl alcohol prior to therapy administration. Only one ear received treatment at a time. Treatment was first applied to the right ear during the first visit, followed by the left ear during the second visit and so on. The experimental objects were replaced every other day, thrice a week (except Sunday) to avoid local irritation of the auricular points under treatment. The total treatment period was six weeks.

#### Treatment effect evaluation

**Fig. 2:**
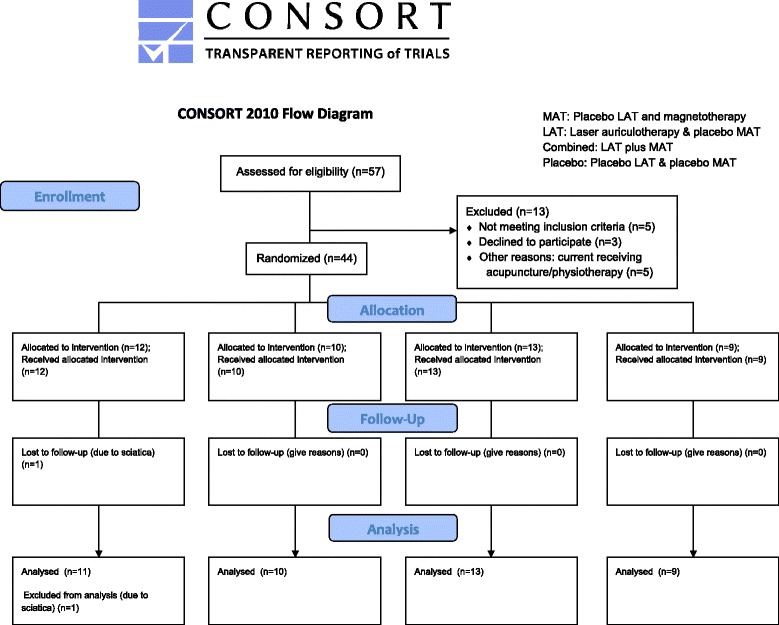
Flow diagram of study. MAT: Placebo LAT and magnetotherapy, LAT: Laser auriculotherapy & placebo MAT, Combined: LAT plus MAT, Placebo: Placebo LAT & placebo MATᅟ

Another researcher who did not know the type of treatment modality received by the participants evaluated the effect of treatment to achieve the effect of evaluator blinding. The flow diagram for this trial is presented in Fig. 2. The effect of treatment was evaluated on the most painful knee if the subject suffered from bilateral OA knee. The numerical rating scale (NRS) of pain was used to evaluate the pain perception of the subjects. The subjects were asked to rate their maximal OA-induced knee pain from 1 to 10 in the recent two to three days; with higher rating indicating greater severity of the pain they experienced [17]. Timed-up-and-go test (TUGT) was performed to assess the ambulation status of the subject. The subject was requested to rise from a chair and walk at a comfortable and safe pace to a destination located 3 m away, turn around, return to the chair and sit down again [18]. A standard goniometer was used to measure the active and passive range of movement (ROM) of the knees during flexion and extension. Subjects’ expectations of the therapy were evaluated [19, 20]. The patients were asked how much they believed the MAT or LAT would help them manage their problem. The satisfaction of the subjects was also evaluated using a 10-point scale, with the higher scores indicating greater satisfaction towards the therapy. We collected socio-demographic characteristics, including age, gender, number of years suffering from OA knee, co-morbidities and current medications. Whether the subjects experienced any itchiness on their auricles induced by the plastic tapes were also evaluated.

### Data analyses

Descriptive statistics, namely, demographic characteristics and mean (SD) of parameters before and after intervention, were computed. Association between categorical variables was examined using *χ*^2-^test or Fisher’s exact test (when the cell count is less than 5). Kruskal–Wallis test was used to evaluate differences among groups. Wilcoxon sign-ranked test was used to examine within-group comparison. Analyses were conducted using SPSS Statistics 20 or R version 3.0.2. (for Fisher’s exact test), and *p* < 0.05 indicated statistical significance.

## Results

### Participants’ characteristics

The study was conducted from January to September 2015. Among the 57 recruited Chinese elderly in total, 13 were excluded because of the following reasons: gout, 4; below 60 years old, 1; recently received acupuncture/physiotherapy, 5; and planning to travel, 3. Recruitment rate was 73.7 %. A total of 44 subjects were randomly divided into four groups (group 1, 12; group 2, 10; group 3, 13; and group 4, 9). Only one participant from group 1 dropped out from the study because of sciatica, resulting in an attrition rate of only 2.38 %. All other participants (*n* = 43) completed the six-week intervention and post-assessment. The participant recruitment CONSORT flow diagram is illustrated in Fig. 2.

No significant difference was found in the demographic and clinical characteristics among the four groups at the baseline (Table 1). The mean age of the 43 participants was 72.7 (SD = 7.04) years old. Majority of the participants were female (*n* = 41). The average number of years with OA knee was 7.82 (SD = 7.07). Over 93 % were suffering from bilateral OA knee problem (*n* = 39) and other comorbidities, such as hypertension or diabetes mellitus (*n* = 39).

**Table 1:** Baseline background and clinical characteristics of the study sample (*n* = 43)

	All (*n* = 43)	Group 1 (*n* = 11)	Group 2 (*n* = 10)	Group 3 (*n* = 13)	Group 4 (*n* = 9)	*p*-value
Age mean (sd)	72.66 (7.04)	73.00 (6.31)	72.80 (6.68)	71.62 (7.75)	73.86 (8.53)	0.839^a^
Gender						
Male	2	2	0	0	0	0.125^b^
Female	41	9	10	13	9	
Number of years with OA knee mean (sd)	7.82 (7.07)	4.95 (4.16)	8.20 (8.13)	9.77 (8.44)	8.14 (8.26)	0.468^a^
Side(s) of OA knee						
Unilateral	4	1	2	0	1	0.429^b^
Bilateral	39	10	8	13	8	
Comorbid illness						
Yes	39	11	8	11	9	0.307^b^
No	4	0	2	2	0	

Group 1 = Placebo LAT & MAT; Group 2 = LAT & placebo MAT; Group 3 = LAT plus MAT; Group 4 = Placebo LAT & Placebo MAT

^a^Kruskal-Wallis test

^b^Fisher’s exact test

### Treatment effect

Preliminary results indicated the absence of differences in the NRS, TUGT and active/passive knee flexion and extension at the baseline, as well as post-therapy, among the four groups (Table 2). Even though the differences of these parameters between groups were not significant, the relative differences of NRS and TUGT in subjects who received combined MAT plus LAT (group 3) were higher than those in other groups (group 1, 2 and 4) (Table 2). In addition, the results of within-group comparison showed that nearly all parameters demonstrated significant differences before and after intervention in subjects who received MAT and/or LAT (namely, groups 1–3), but no differences were observed in the placebo group (Group 4) (Table 3).

**Table 2:** Outcome variables across four groups before and after intervention^a^

	All (*n* = 43)	Group 1 (*n* = 11)	Group 2 (*n* = 10)	Group 3 (*n* = 13)	Group 4 (*n* = 9)	*p*-value
Numerical rating scale						
Baseline	6.61 (1.63)	6.64 (1.69)	7.00 (1.63)	6.69 (1.80)	5.86 (1.22)	0.621
Post intervention	4.73 (2.04)	5.27 (1.79)	4.60 (1.90)	4.15 (2.12)	5.14 (2.55)	0.579
Relative difference	−1.88 (2.14)	−1.37 (1.36)	−2.40 (1.65)	−2.54 (2.47)	−0.71 (2.75)	0.299
Timed-up and-go test						
Baseline	18.25 (5.05)	18.14 (3.98)	16.31 (3.42)	20.16 (5.67)	17.64 (6.85)	0.243
Post intervention	15.31 (5.26)	15.39 (4.66)	15.14 (5.20)	14.99 (4.66)	16.04 (7.96)	0.985
Relative difference	−2.93 (4.05)	−2.75 (2.61)	−1.17 (4.24)	−5.16 (4.75)	−1.60 (2.81)	0.132
Knee flexion (active)						
Baseline	115.32 (15.34)	118.18 (13.36)	109.70 (13.43)	113.38 (17.54)	122.43 (15.79)	0.524
Post intervention	126.54 (11.67)	130.82 (10.20)	123.30 (8.42)	125.23 (13.24)	126.86 (14.92)	0.452
Relative difference	11.22 (12.09)	12.64 (11.99)	13.60 (15.96)	11.85 (9.38)	4.43 (10.42)	0.554
Knee flexion (passive)						
Baseline	122.54 (11.45)	125.27 (11.43)	120.50 (9.58)	119.38 (14.06)	127.00 (7.62)	0.536
Post intervention	131.46 (10.80)	135.73 (10.26)	129.40 (6.08)	128.77 (11.75)	132.71 (14.59)	0.372
Relative difference	8.93 (8.34)	10.45 (7.67)	8.90 (9.75)	9.38 (5.53)	5.71 (12.04)	0.611
Knee extension (active)						
Baseline	−4.61 (4.01)	−4.73 (5.00)	−4.50 (3.03)	−4.38 (4.33)	−5.00 (3.74)	0.944
Post intervention	−2.93 (2.91)	−3.00 (2.93)	−3.10 (2.51)	−2.77 (3.22)	−2.86 (3.44)	0.979
Relative difference	1.68 (3.33)	1.40 (2.41)	1.40 (2.41)	1.62 (3.66)	2.14 (2.91)	0.996
Knee extension (passive)						
Baseline	−1.73 (4.51)	−1.82 (4.24)	−1.80 (4.02)	−2.00 (5.87)	−1.00 (3.42)	0.939
Post intervention	0.00 (3.23)	0.09 (2.88)	0.20 (3.19)	0.08 (3.12)	−0.57 (4.54)	0.980
Relative difference	1.73 (3.69)	1.91 (2.39)	2.00 (4.16)	2.08 (4.87)	0.43 (2.37)	0.801

Group 1 = Placebo LAT & MAT; Group 2 = LAT & placebo MAT; Group 3 = LAT plus MAT; Group 4 = Placebo LAT & Placebo MAT

^a^Between group comparisons were made using Kruskal-Wallis test

**Table 3:** Within group comparison of different outcome variables

	Baseline	Post-intervention	*p-*value (within group comparison to the baseline)^a^
Group 1 (*n* = 11)			
Numerical rating scale	6.64 (1.69)	5.27 (1.79)	0.017*
Timed-up and-go test	18.14 (3.98)	15.39 (4.66)	0.013*
Knee flexion (active)	118.18 (13.36)	130.82 (10.20)	0.005**
Knee flexion (passive)	125.27 (11.43)	135.73 (10.26)	0.004**
Knee extension (active)	−4.73 (5.00)	−3.00 (2.93)	0.107
Knee extension (passive)	−1.82 (4.24)	0.09 (2.88)	0.027*
Group 2 (n-10)			
Numerical rating scale	7.00 (1.63)	4.60 (1.897)	0.008**
Timed-up and-go test	16.31 (3.42)	15.14 (5.20)	0.241
Knee flexion (active)	109.70 (13.43)	123.30 (8.42)	0.032*
Knee flexion (passive)	120.50 (9.58)	129.40 (6.08)	0.007**
Knee extension (active)	−4.50 (3.03)	−3.10 (2.51)	0.121
Knee extension (passive)	−1.80 (4.02)	0.20 (3.19)	0.152
Group 3 (*n* = 13)			
Numerical rating scale	6.69 (1.80)	4.15 (2.12)	0.005**
Timed-up and-go test	20.16 (5.67)	14.99 (4.66)	0.005**
Knee flexion (active)	113.38 (17.54)	125.23 (13.24)	0.002**
Knee flexion (passive)	119.38 (14.06)	128.77 (11.75)	0.002**
Knee extension (active)	−4.38 (4.33)	−2.77 (3.22)	0.182
Knee extension (passive)	−2.00 (5.87)	0.08 (3.12)	0.153
Group 4 (*n* = 9)			
Numerical rating scale	5.86 (1.22)	5.14 (2.55)	0.343
Timed-up and-go test	17.64 (6.85)	16.04 (7.96)	0.176
Knee flexion (active)	122.43 (15.79)	126.86 (14.92)	0.176
Knee flexion (passive)	127.00 (7.62)	132.71 (14.59)	0.225
Knee extension (active)	−5.00 (3.74)	−2.86 (3.44)	0.058
Knee extension (passive)	−1.00 (3.42)	−0.57 (4.54)	0.605

Group 1 = Placebo LAT & MAT; Group 2 = LAT & placebo MAT; Group 3 = LAT plus MAT; Group 4 = Placebo LAT & Placebo MAT

*Statistically significance *p* < 0.05

**Highly statistically significant *p* < 0.001

^a^Within group comparisons were made using Wilcoxon Signed ranks test

Relative differences in NRS scores were *μ*_1_ = −1.37, *μ*_2_ = −2.40, *μ*_3_ = −2.54 and *μ*_4_ = −0.71 in the MAT, LAT, combined and placebo groups, respectively, with SD of = 2.14. This effect corresponds to a Cohen’s *f* of 0.319 (intermediate effect) [21]. Only 11.6 % (*n* = 5) of the participants reported itchiness on their auricles induced by the plastic tapes that adhered to the acupoints, but the symptoms were resolved automatically after the plasters were removed.

The participants generally exhibited strong faith in complementary therapies (mean = 7.33). Prior to the intervention, the expected treatment effect towards MAT, LAT and both approaches (as expressed by the participants) were 8.00, 7.95 and 8.53, respectively. After the intervention, satisfaction toward therapy was high across groups (mean = 8.70), and more than 95 % (*n* = 41) of the participants indicated that they would recommend this therapy to others.

## Discussion

AT can be applied to elders with OA knee. The overall satisfaction of participants of all groups towards the therapy was high with an extremely low attrition rate (2.38 %). The positive impression towards the therapy indirectly reflected that blinding was successful among the subjects because of the successful application of placebo in different groupings. The subjects were debriefed regarding the treatment they received upon study completion. The low attrition rate is also probably due to the fact that the targeted population was already retired and had more free time to participate in the activities of the elderly centres. Many of the subjects did not receive any active treatment for OA knee and agreed to try a non-traumatic alternative therapy. The participants generally expressed strong faith in complementary therapies, with high expectation of the treatment’s effect. The high satisfaction towards therapy across groups, including the subjects receiving placebo treatment, may indicate that subject blinding was successfully achieved.

Quality assurance of the study, such as intensive coaching given to the therapist, was observed to ensure the accuracy of the identification of ear acupoints. Interrater reliability of the evaluators involved in the assessment was established. All research personnel involved in the study had to follow the “Good Clinical Practice” guidelines to protect human subjects and to collect quality data [22]. The successful blinding of evaluators who did not know the type of treatment modality received by the participants indicated that the measurements were objective and were obtained without personal bias.

The effect of treatment was evaluated not only by subjective pain scale but also by adopting objective measures, such as TUGT and goniometer measurements, to evaluate the active and passive ROM of the knees during flexion and extension. Preliminary results indicated the absence of differences in the NRS, TUGT and active/passive knee flexion and extension post-therapy among the four groups. These differences are probably due to the small number of participants enrolled in this study. Even though the differences of these parameters between groups were not significant, the relative differences of NRS (pain relief) and TUGT (ambulation status) in subjects who received combined MAT plus LAT were higher than those treated with MAT or LAT alone, or the placebo group. In addition, results of within-group comparison illustrated that nearly all parameters demonstrated significant differences before and after the intervention in subjects receiving MAT and/or LAT, whereas no differences could be seen in the placebo group. The effectiveness of magnetic pellets have been demonstrated in a number of experimental studies with promising effect probably because of their association with the interaction of magnetic fields with tissues that result in functional changes in the body [10, 11, 23],. Preliminary findings of this study indicated that using combined MAT plus LAT exerted a relatively better effect than using MAT or LAT alone. This result agrees with the synergistic effect of combined therapeutic laser and herbal medication protocols on injured medial collateral ligaments (MCLs) of rat knees [24]. The combined protocols further enhanced the biomechanical properties of repairing rat MCLs in comparison with separate applications [24]. The use of laser beam was associated with the release of endorphins, which exert a transquilising effect on the patient during addiction therapy [25]. Endorphins are natural pain killers of the body [26]. The therapeutic effect in the current study could be optimised by the application of MAT after LAT because the former approach could offer continuous stimulation of acupoints after laser treatment, as long as the magnet pellets on the ears are in situ.

### Limitations and recommendations of study

Generalisation of results is limited because of the small number of participants enrolled in this feasibility study. A larger sample size with more male subjects should be considered in future trials so as to evaluate whether any gender difference affects the treatment effect. Other measures that indicate clinical relevance could be considered in future studies, including the disease-specific instrument using Western Ontario and McMaster Universities Osteoarthritis Index (WOMAC), quality of life measurement and the health resource utilisation using cost-effectiveness analysis. Moreover, longer follow up, that is, a minimum of six months of the therapy, may be considered in evaluating the sustained treatment effect.

## Conclusion

This study demonstrates that the AT protocol adopted in this study for elders with OA knee is feasible and could be applied in future larger-scale study. The low attrition rate in this study was obtained. Successful blinding in subjects and evaluators was achieved. Subjects who received combined MAT plus LAT exhibited stronger treatment effect in terms of pain relief, ambulation status and range of movement of the knees during flexion and extension compared with those treated with MAT or LAT alone or compared with the placebo group. Larger sample size should be considered in the future trial to determine the causal relationship between treatment and effect.

## Abbreviations

LAT, laser auriculotherapy; MAT, magneto-auriculotherapy; NRS, Numerical rating scale; OA, osteoarthritis; ROM, range of movement; TCM, traditional Chinese medicine; TUGT, Timed-up-and-go test
