# Erratum to: Using auriculotherapy for osteoarthritic knee among elders: a double-blinded randomised feasibility study

**DOI:** 10.1186/s12906-016-1370-z

**Published:** 2016-10-12

**Authors:** Lorna K. P. Suen, Chao Hsing Yeh, Simon K. W. Yeung

**Affiliations:** 1 School of Nursing, The Hong Kong Polytechnic University, HungHom, Hong Kong; 2 Johns Hopkins School of Nursing, Room 421, 525, N. Wolfe Street, Baltimore, MD21205 USA

## Erratum

Following publication of the original article [1] it was brought to our attention that a mistake was overlooked during the proofing process. In the ‘Authors’ information’ section, it is said that the second author is an associate professor of the School of Nursing, The University of Pittsburgh, USA. However, this should instead read as Johns Hopkins School of Nursing and not The University of Pittsburgh. The authors would like to apologise for any confusion caused.
